# Conculcation or Inoculation?

**Published:** 1895-01-12

**Authors:** 


### The Hospital

An Institutional Journal of

The Medical Sciences and Hospital Administration, with

AN EXTRA NURSING SUPPLEMENT.

Vol. XVII.—No. 433.] [Jan. 12, 1895.

### Conculcation or Inoculation?

At what stage of his experience is a man entitled to demand admission for a new word into his mother tongue? That, no doubt, depends upon the mother tongue—whether it be an ancient language of majestic history, or a savage speech in which new ideas can by no possibility be adequately expressed. Sir Benjamin Richardson asks the medical profession to accept the word "conculcation" as a kind of philosophico-medical antithesis to "inoculation." Perhaps Sir Benjamin has as good a right to coin a new, or half-new, word as most of the medical writers who have added barbarisms to the dialect of medical science. Be that as it may, he has introduced the word; the *Times* has accepted and published it without comment; and it must, we suppose, be now generally accepted for the old and frequently invoked reason that we do not happen to possess a better.

It is the future of medical progress which Sir Benjamin Richardson is concerning himself about. In his capacity of president of the Sanitary Inspectors' Association he delivered his inaugural address the other day, and among other things he set "conculcation" over against "inoculation" as a mode of medical and sanitary progress; and he threw the weight of his own individual judgment very emphatically into the scale which favoured conculcation. What then is "conculcation" as a *modus medicinæ*? What inoculation is we do not require to ask. Conculcation, to use Sir Benjamin's own definition, consists in "the one grand element of perfect cleanliness in every nation, every family, every individual." That is the old and tried method of sanitary progress; and that, much more than inoculation, is the god of this venerable sanitarian's sanitary idolatry. He speaks, indeed, of inoculation with small respect. Is it worthy of being called a method of sanitation or disease prevention at all, he asks? It "pretends to render the body immune against diseases," he says; and it "predicts the greatest triumphs for its future." But at best it only professes to "prevent one disease by producing another." It appears to Sir Benjamin to be a method by which, whatever else happens, "disease" in some of its forms must be "eternally perpetuated." Whilst in the way of advantage the most that can be hoped for from it is a "temporary remission of some diseases with the corresponding admission of others to take their place."

Here is a gauntlet which we are glad to see thrown down. We shall be equally pleased to see the challenge promptly accepted by the other side. A competent discussion of this and kindred subjects, as we plainly stated in our critical review of the *Annus Medicus* 1894, is what is now demanded above all other things. Is conculcation, or perfect cleanliness in every detail of national and individual life, the true method of sanitary progress? Or is inoculation, and consequent immunisation against disease, the true and proper method? Let the scientific gladiators join issue on this great international and world-wide question. It can be decided by two methods only—the methods of an adequate accumulation of facts, and a competent chain of reasoning. The facts will not, as many crude young laboratory doctors think, stand without the reasoning; still less will the reasoning stand without the facts. Both are entirely indispensable for a scientific demonstration which aspires to be permanent.

But we would like to whisper a suggestion into the ears of both classes of sanitarians. It is this: Is there any natural and necessary antagonism between these two methods of sanitary progress? Supposing we agree, as we all shall agree, that perfect cleanliness is at least one of the most important factors which go to the making up of perfect health, is there any reason in that why we should not also make our bodies completely "immune" against all bacterial diseases if we can? Cleanliness, even in the most highly civilised of communities, is, and must for ever remain, quite a relative factor. It can never be absolute. When a nation shall have become as clean as it can possibly be made, it will still have to deal in a practical way with manifold varieties of accumulated filth: and every variety of filth is a genderer of diseases. Here then is the opportunity of the inoculator.

The real point for sanitarians to consider is, not whether "stamping out" on the one hand, or "stamping in" on the other, is the more philosophical and the more practically serviceable method of sanitation; but whether, after the highest possible degree of national and individual cleanliness has been reached, we cannot find in some forms of inoculation useful adjuncts towards the still more perfect suppression and extinction of every form of bacillary disease. That is the true goal at which investigators must aim. We must not divide ourselves into opposite schools, unless it be by mutual agreement for the better elucidation of the truth. True science knows but one school, the school of inquiry and experimental proof. What we must bear in mind at the present moment is, that inoculation has not been tried and proved as cleanliness has been tried and proved; but that it is at the very beginning and initial stage of what may prove to be a disappointing failure or a brilliant and permanent success. Give it an abundance of time and opportunity, and we, or our descendants, will know how to appraise its worth.

